# Aspects cliniques de la Neurofibromatose de type 1 vue au service de Dermatologie Du Centre Hospitalier Universitaire Antananarivo, Madagascar

**DOI:** 10.48327/mtsi.v2i2.2022.247

**Published:** 2022-05-27

**Authors:** Fandresena Arilala SENDRASOA, Aurélie RASOARISATA, Lala Soavina RAMAROZATOVO, Fahafahantsoa RAPELANORO RABENJA

**Affiliations:** Département de dermatologie, Centre hospitalier universitaire Joseph Raseta Befelatanana, Antananarivo, Madagascar

**Keywords:** Neurofibromatose type 1, Forme sporadique, Antananarivo, Madagascar, Océan Indien, Neurofibromatosis type 1, Sporadic forms, Antananarivo, Madagascar, Indian Ocean

## Abstract

**Introduction:**

La neurofibromatose type 1 (NF1) est une maladie autosomique dominante, à expression multisystémique. Sa prévalence varie selon les pays et peu de données sur la NF1 sont connues à Madagascar.

**Méthodologie:**

Une étude transversale rétrospective a été réalisée au service de dermatologie du Centre hospitalier universitaire Joseph Raseta Befelatanana d'Antananarivo, Madagascar, sur une période de 6 ans (de janvier 2014 à décembre 2019). Ont été inclus dans cette étude les patients vus en consultation porteurs de NF1 dont le diagnostic a été confirmé et chez lesquels une enquête généalogique a pu être faite.

**Résultats:**

Vingt-huit cas de neurofibromatose ont été inclus avec un sexe-ratio H/F de 0,87. L’âge moyen était de 24 ans avec des extrêmes de 11 et 54 ans. La tranche d’âge 16-35 ans était la plus touchée. Dix-sept patients présentaient la forme sporadique. Tous les patients présentaient des taches café au lait et des neurofibromes cutanés, 15 patients portaient des nodules iridiens de Lisch. Les difficultés d'apprentissage, l’épilepsie et la scoliose étaient les complications les plus représentées.

**Conclusion:**

Les manifestations cliniques de la NF1 sont extrêmement variables. Même si les complications systémiques sont rares, le suivi des patients est indispensable.

## Abstract (en)

**Introduction:**

Neurofibromatosis 1 (NF1) is an inherited disease, in an autosomal dominant manner, with complex multi-system involvements. Prevalence varies from one country to another. However, little is known about neurofibromatosis in African countries, particularly in Madagascar.

**Methodology:**

A descriptive retrospective study from 2014 to 2019 was conducted at the service of dermatology at University Hospital Joseph Raseta Befelatanana in Antananarivo, including all patients with neurofibromatosis according to National Institutes of Health Consensus Conference criteria for whom genealogical investigation could be made.

**Results:**

Among 32 cases of NF1 seen during 6 years, 28 cases were included with a sex ratio M/F of 0.87. The mean age was 24 years ranging from 11 to 54 years. Seventeen patients presented sporadic forms. All patients had “café au lait” spots and cutaneous neurofibromatosis. Three cases presented plexiform neurofibromas which cause significant cosmetic and functional problems by their size and their displayed topography. Fifteen patients had Lisch nodules but no case of optic glioma was identified. Neurological symptoms such as learning difficulties, epilepsy and headache were frequent in our case series. However, access to medical imaging was very limited. Scoliosis was the most common orthopedic complication.

**Conclusion:**

The clinical manifestations of NF1 are extremely variable. Although the possibility of systemic complications seems to be low, patients must be followed up.

## Introduction

La neurofibromatose de type 1 (NF1) est une génodermatose à expression multisystémique qui atteint préférentiellement les cellules dérivées de la crête neurale [17]. C'est la neurofibromatose la plus fréquente, avec une incidence estimée d'environ 1/2 000 et une prévalence de 1/2 000 à 1/3 000 dans une série d’études européennes, nord-américaines et océaniennes [12, 22]. L'espérance de vie moyenne des patients atteints de NF1 est réduite approximativement de8à20 ans par rapport à la population générale [22, 19]. La NF1 est caractérisée par des manifestations cutanées à type de taches café au lait, lentigines et neurofibromes, auxquelles peuvent être associées d'autres manifestations telles l'atteinte oculaire (nodules iridiens de Lisch), osseuse, neurologique, orthopédique et gastro-intestinale [9]. Le terme neurofibromatose a été introduit en 1882 par Friedrich von Recklinghausen [24].

L'objectif de ce travail est de décrire le profil épidémiologique de la neurofibromatose chez des patients malgaches et d’établir l'arbre généalogique de chaque patient afin d’évaluer la transmission génétique de cette maladie.

## Méthodologie

Une étude transversale rétrospective a été réalisée au service de dermatologie du Centre hospitalier universitaire Joseph Raseta Befelatanana à Antananarivo (Madagascar) sur une période de 6 ans (de janvier 2014 à décembre 2019).

Ont été inclus dans cette étude les patients vus en consultation porteurs de NF1 dont le diagnostic a été confirmé sur les critères diagnostiques de la Conférence de consensus tenue à Bethesda en 1987 [13] et chez lesquels une enquête généalogique a pu être réalisée.

### Analyse statistique

Les données ont été enregistrées sur le logiciel Excel^®^. Pour l'analyse statistique, le logiciel Epi-info Version 7.2.3.1 a été utilisé.

### Considérations éthiques

La confidentialité a été respectée pour tous nos patients. Les photos et les autres matériels relatifs à notre étude sont anonymes et ont été manipulés en respectant rigoureusement cet anonymat.

## RéSultats

Trente-deux cas de neurofibromatose ont été collectés en 6 ans parmi les 8301 patients vus en consultation de dermatologie, soit une proportion de 0,39 %. Vingt-huit patients ont été inclus dans cette étude. Quatre patients ont été exclus en raison de données insuffisantes dans les dossiers.

L’âge moyen des patients au moment du diagnostic a été de 24 ans avec des extrêmes de 11 et 54 ans. La tranche d’âge la plus touchée a été celle de 16-35 ans, soit 78 % des cas. Une prédominance féminine a été trouvée dans notre étude (13 hommes/15 femmes), soit un sexe-ratio de 0,87.

La neurofibromatose est apparue à la naissance chez 8 patients, dans la petite enfance chez 14 patients et à l'adolescence chez 6 patients. Onze cas ont déclaré une atteinte d'un parent de premier degré et dans 17 cas il s'agissait d'une néomutation, soit respectivement 39 % et 61 %. Aucune notion de lien consanguin objectif n'a été trouvée chez les patients. En étudiant les arbres généalogiques des patients, l'origine familiale de la maladie a été retrouvée chez 11 cas (5 origines maternelles et 6 paternelles). Deux patients avaient des sœurs atteintes.

Cette analyse a porté sur 26 patients non apparentés et un père et son fils. Ce qui donne au total un nombre de 27 familles examinées. Quarante-six membres de ces familles sont porteurs de NF1. Le nombre de cas de néomutation est de 17. Le rapport mutation-hérédité est égal à 0,37. L’âge moyen des patients lors de l'apparition phénotypique des néomutations a été de 20 ans.

Le tableau I montre la répartition des patients inclus selon les données démographiques et cliniques.

**Tableau I:** Répartition des patients inclus selon les données démographiques, cliniques et paracliniques Distribution of participants according to demographic, clinical and paraclinical characteristics

Caractéristiques	N
**Âge (ans)**	
< 16	3
[16-35]	22
< 35	3
**Genre**	
masculin	13
féminin	15
**Âge (ans) de début de la neurofibromatose**	
[0-1]	8
[1-10]	14
< 10	6
	
Signes cutanés de la neurofibromatose	
taches café au lait	28
lentigines	20
neurofibromes cutanés	28
neurofibromes sous-cutanés	7
neurofibromes plexiformes	3
	
Manifestations neurologiques	
troubles neurocognitifs	13
céphalées	7
convulsions	4
**Signes oculaires (nodules de Lisch)**	15
**Signes osseux**	10

### Signes cutanés

Les taches café au lait (TCL) et les neurofibromes ont été présents chez tous nos patients. Les TCL sont apparues à un âge moyen de 4,6 ans. Quatorze patients de notre étude ont eu des TCL avant l’âge de 3 ans. Douze TCL caractéristiques en moyenne ont été trouvées chez chaque patient. La localisation des TCL sur le tronc a été trouvée chez tous les patients et sur les membres chez 13 cas. Des TCL au niveau céphalique ont été présentes chez 3 cas.

Les neurofibromes sont apparus à un âge moyen de 14 ans. Les neurofibromes cutanés ont été présents dans tous les cas, tous âges confondus, n'apparaissant qu’à la puberté. Les neurofibromes sous-cutanés ou nodulaires ont été trouvés chez 7 cas. Les neurofibromes plexiformes ont été présents chez 3 cas, ayant posé des problèmes esthétiques majeurs et fonctionnels par leur taille et leurs situations particulières au niveau de la face, dos, bras et face postérieure de la cuisse.

Les lentigines axillaires et/ou inguinales ont été retrouvées chez 20 patients. Ces lentigines apparaissent souvent après les TCL avec un âge moyen d'apparition de 5,15 ans. Leur localisation préférentielle était axillaire chez 26 cas.

Le tableau II montre la répartition des patients selon la localisation des signes cutanés.

**Tableau II:** Répartition des patients selon la localisation des signes cutanés Distribution of patients according to the topography of cutaneous signs

Signes cutanés	Localisation			
	Tête	Tronc	Membres
Taches café au lait	3	28	13
Neurofibromes cutanés	15	28	20

**Figure 1:**
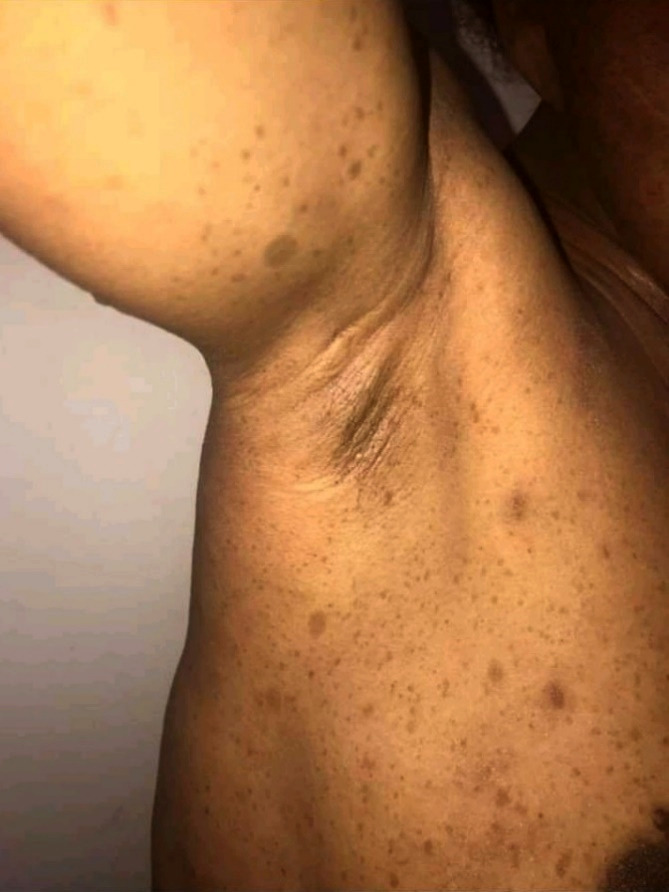
“Café au lait” spots and axillary lentigines Taches café au lait et lentigines axillaires

**Figure 2:**
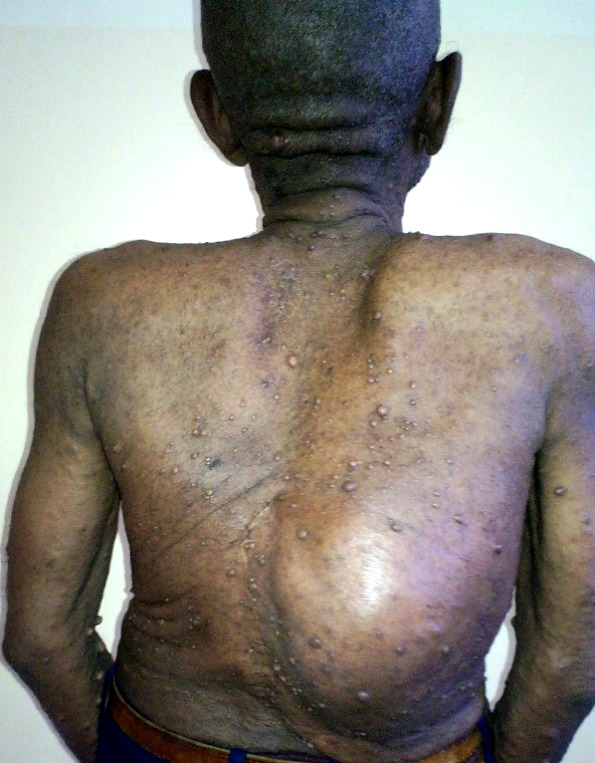
Cutaneous neurofibromas and a plexiform neurofibroma of the lumbar region Neurofibromes cutanés et un neurofibrome plexiforme de localisation lombaire

La figure 1 montre des taches café au lait et des lentigines axillaires chez un homme de 47 ans. La figure 2 montre des neurofibromes cutanés et un neurofibrome plexiforme de localisation lombaire.

### Signes oculaires

Sur les 28 patients atteints de NF1, 16 ont pu faire un examen à la lampe à fente : 15 ont eu des nodules de Lisch, bilatéraux dans 10 cas. Aucun cas de gliome des voies optiques n'a été identifié.

### Signes osseux

Des lésions osseuses et des complications relatives ont été notées chez 11 patients : 6 cas de scoliose dorso-lombaire, 2 cas de cyphoscoliose, 1 cas de pseudarthrose et 2 cas de dysplasie congénitale de la jambe. Aucun cas de dysplasie des ailes sphénoïdales n'a été vu dans notre étude.

### Manifestations neurologiques

Vingt-quatre patients de cette étude ont eu des atteintes neurologiques. Des céphalées de type migraineux ont été rapportées dans 7 cas. Des crises convulsives dans la petite enfance ont été notées chez 4 patients. Des troubles neurocognitifs ont été objectivés chez 13 cas, représentés essentiellement par des difficultés d'apprentissage avec un retard scolaire chez 3 cas, des troubles de vigilance, de concentration, et des troubles mnésiques dans 3 cas.

Treize patients ont pu effectuer un électro-encéphalogramme, 9 tracés (69%) ont été normaux et 4 tracés ont été de type épileptiforme. Deux patients ont fait une imagerie cérébrale (un patient a fait une tomodensitométrie cérébrale qui a montré une dilatation ventriculaire minime en rapport avec une hydrocéphalie et une atiente a fait une imagerie par résonance magnétique cérébrale et des voies optiques qui n'a trouvé aucune anomalie).

## Discussion

Peu de données concernant la neurofibromatose en Afrique et dans l'Océan indien ont été rapportées, notamment à Madagascar, en dehors de quelques séries de cas décrites en Afrique du Sud et au Nigéria [14, 18]. Selon notre étude, la neurofibromatose est une pathologie relativement rare, 5 cas par an ont été vus, représentant 0,39 % de l'ensemble des consultations dermatologiques. Notre étude a également montré que la neurofibromatose survient dans 50 % des cas dans la petite enfance. Quant à l'enquête généalogique, elle indique un rapport mutation-hérédité est de 0,37. Le diagnostic de la neurofibromatose est souvent tardif bien qu'il soit aisé, nécessitant donc un effort important de sensibilisation aussi bien auprès des médecins que dans la population générale.

La prédominance féminine constatée dans cette étude (sexe-ratio H/F = 0,87) a été similaire à celle qui est trouvée dans la littérature [5, 21]. Elle pourrait s'expliquer par l'aspect inesthétique des lésions qui incite les femmes à consulter. Pourtant, une prédominance masculine a été trouvée dans l’étude menée par Odebode *et al.* au Nigéria [14].

Dans cette présente étude, 11 cas étaient des formes familiales et 17 cas des néomutations. Ceci mérite de ne pas sous-estimer la possibilité d'un diagnostic de NF1 même en l'absence de forme familiale identifiée. Il existe une prédominance des formes sporadiques dans notre série, alors que la littérature décrit 50 % de formes familiales et 50 % de formes sporadiques [10]. Routier *et al.* ont rapporté dans leur série que la NF1 était héritée dans 68 % des cas [20]. La grande variabilité des manifestations cliniques de la NF1 peut faire méconnaître la présence d'antécédents familiaux [9, 15]. Les membres de la famille des patients atteints des formes sporadiques sont asymptomatiques. Cependant, l'enquête familiale complète avec examen ophtalmologique des membres ascendants au premier degré des patients n'a pas pu être réalisée du fait de la dispersion géographique des familles.

Les TCL et les neurofibromes étaient présents chez tous les patients. Cela est peut-être dû au fait que les TCL sont les premiers signes de la maladie et que les patients consultent le plus souvent après l'apparition des neurofibromes ou au moment où ceux-ci deviennent gênants. Pourtant, Ramanjam *et al.* n'ont trouvé des neurofibromes que chez 4 % des patients dans leur série pédiatrique sud-africaine (enfants < 12 ans) [18]. Les TCL sont apparues dès la naissance chez 14 cas. Ces résultats ont été justifiés par des études portant sur l’âge d'apparition des TCL : le plus souvent dès la naissance ou durant les 2 premières années de la vie [16]. Leur nombre suffisant (au moins 6 TCL) chez tous les patients est conforme aux données des différentes études [16]. Le tronc a été la localisation la plus représentée des TCL selon certains auteurs [15].

L’âge moyen d'apparition des neurofibromes était de 14 ans. D'autres études ont trouvé des résultats similaires [15]. Les neurofibromes débutent à la puberté et ils sont exceptionnellement absents à l’âge adulte. Selon d'autres études, les neurofibromes apparaissent généralement pendant la préadolescence [19]. La localisation des neurofibromes sur le tronc était plus fréquente. Comme la nôtre, d'autres études expriment que le siège principal des neurofibromes était le tronc mais les autres parties du corps ne sont pas épargnées [15]. Au Nigéria, Odebode *et al.* ont décrit une localisation fréquente des neurofibromes au niveau des extrémités [14]. Les neurofibromes cutanés qui apparaissent en général à l'adolescence ont souvent de lourdes conséquences psychologiques et sociales par leur aspect visuel parfois spectaculaire [6].

Les neurofibromes plexiformes sont présents chez 3 cas, ce qui est inférieur à ce qu'on retrouve dans la littérature (16 à 26 % des cas) [4, 18]. Ils sont accompagnés de dysplasie à la fois cutanée, musculaire et également osseuse. Ces neurofibromes plexiformes peuvent être responsables de complications esthétiques majeures (hypertrophie de segments corporels), avec parfois une atteinte ophtalmologique (amblyopie) quand ils siègent sur la face. Ces lésions sont responsables d'un préjudice esthétique justifiant une orientation spécifique [4].

Les nodules de Lisch sont présents chez 15 patients (53%) de notre étude, ce qui coïncide respectivement au résultat de l'ordre de 17 % à 52 % des patients des séries de Kim *et al.* [11]. Leur présence reste un bon critère diagnostique et leur absence n’élimine pas le diagnostic de NF1. Dans notre étude, ces nodules de Lisch sont bilatéraux dans 10 cas, représentant ainsi un véritable critère diagnostique quasi pathognomonique de la NF1 contre 5 cas de nodules de Lisch unilatéraux.

Le gliome des voies optiques est la tumeur la plus fréquente au cours de la NF1, avec une incidence variable de 1,5 à 70 % selon les séries [2, 3, 8], aucun cas n'a été noté dans notre série. Ceci est à pondérer car une exploration radiologique chez les patients n'a pas pu être effectuée.

En 2013, Ferner *et al.* ont rapporté que les principales complications chez les enfants de moins de 16 ans étaient les difficultés d'apprentissage, lesquelles constituent un problème majeur au cours de la NF1 [6, 7]. Elles sont remarquables par leur fréquence qui varie de 20 à 60 % et par leurs aspects atypiques [1]. Ces troubles neurocognitifs altèrent parfois considérablement la scolarité, alors que les retards mentaux proprement dits sont de fréquence comparable à celle de la population générale. Dans notre étude, les difficultés d'apprentissage ont été présentes chez 46 % des patients, ce qui est concordant avec les données de la littérature [1], mais inférieur à la fréquence de 70 % dans une étude faite chez les populations pédiatriques en Afrique du Sud [18]. Leurs conséquences les plus marquées étaient un trouble de la mémoire qui entraîne des difficultés de raisonnement, des sorties du circuit scolaire et l'arrêt précoce de la scolarité. Des évaluations de dépistage neuropsychologiques tôt dans la vie et une prise en charge précoce et adaptée pourraient éviter ce genre de complications. Les patients ayant un niveau cognitif normal et qui ne présentent pas de troubles cognitifs ou de troubles d'apprentissage ne devraient pas être négligés car ceux-ci peuvent survenir à tout moment.

La fréquence de l’épilepsie était de 14 % des cas dans notre série, c'est-à-dire supérieure à celle retrouvée dans la littérature qui est de 3,8 % à 7 % [8, 23]. Ce taux élevé peut être expliqué par la plus grande attention donnée par les praticiens pour la recherche des atteintes neurologiques et la pratique systématique d'un électro-encéphalogramme. L'accès à l'imagerie cérébrale reste faible dans notre série de cas, étant donné le coût élevé de ces examens.

Dans notre série, les stratégies diagnostiques comportant la réalisation des principaux examens complémentaires sont conformes aux recommandations actuelles. Les premières consultations ont été d'une importance capitale dans la prise en charge de nos patients. Elles ont permis de prendre en compte l'histoire personnelle et familiale de la maladie, couplée à un examen clinique rigoureux, dermatologique et général. Le diagnostic de la maladie a été essentiellement clinique. Les premières consultations ont également permis de prescrire les bilans d'exploration et d’établir une enquête familiale. En fonction des résultats cliniques et complémentaires de première intention, un certain nombre d'examens plus poussés ont été pratiqués. Une surveillance de l’évolution de la maladie selon la décision de la consultation multidisciplinaire est organisée. Le fait de ne pas revoir certains patients avec leurs résultats est peut-être dû à un problème financier. Malheureusement, quand les patients sont informés de l'absence de traitement spécifique pour leur maladie, ils n’éprouvent plus le besoin de revenir. Or, il est nécessaire de faire un suivi pour dépister précocement des complications.

## Conclusion

Cette étude rétrospective de neurofibromatose au service de dermatologie du Centre hospitalier universitaire de Befelatanana entre janvier 2014 et décembre 2019 a permis de montrer in taux de prévalence de la neurofibromatose de 0,39 %. La tranche d’âge 16-35 ans a été la plus touchée. Le genre féminin a été le plus affecté. Le rapport mutation-hérédité a été inférieur à 1. La majorité des patients ont consulté à cause du préjudice esthétique. Les TCL et les neurofibromes étaient les manifestations cutanées les plus représentées. Les difficultés d'apprentissage, l’épilepsie et la scoliose sont les complications les plus représentées. La particularité de cette étude réside en un taux de néomutation élevé.

## Liens D'intérêts

Les auteurs ne déclarent aucun lien d'intérêt.

## Contribution Des Auteurs

Conception de l’étude : Fandresena Arilala SENDRASOA Réalisation de tout ou partie de l’étude : Fandresena Arilala SENDRASOA, Aurélie RASOARISATA Supervision : Lala Soavina RAMAROZATOVO, Fahafahantsoa RAPELANORO RABENJA Analyse des données : Fandresena Arilala SENDRASOA, Aurélie RASOARISATA Rédaction du manuscrit : Fandresena Arilala SENDRASOA, Aurélie RASOARISATA Relecture et validation du manuscrit : Lala Soavina RAMAROZATOVO, Fahafahantsoa RAPELANORO RABENJA
